# P-172. Assessment of the HIV Case Epidemiological Surveillance System in Sughd Region, Tajikistan, 2024 year

**DOI:** 10.1093/ofid/ofae631.377

**Published:** 2025-01-29

**Authors:** Muhiddin Shamsudinov, Rajabali Sharifov, Roberta Horth, Dilyara Nabirova, Zulfiya H Tilloeva, Gaukhar Mergenova

**Affiliations:** Sughd Regional Center for Prevention and Control of AIDS, Khujand, Sughd, Tajikistan; Central Asia Region FETP, Dushanbe, Dushanbe, Tajikistan; US Centers for Disease Control and Prevention, Dulles, Virginia; CDC Central Asia office, Almaty, Almaty, Kazakhstan; City Disinfection Station, Dushanbe, Republic of Tajikistan, Dushanbe, Dushanbe, Tajikistan; Asfendiyarov Kazakh National Medical University, Almaty, Almaty, Kazakhstan

## Abstract

**Background:**

In recent times, the HIV epidemic has emerged as a major challenge in contemporary healthcare, leading to the demise of 40.4 million individuals globally. Across the timeline of the ailment, fatalities have been recorded, and the spread of the virus persists worldwide, Tajikistan included, where over 10.5 thousand cases have been documented. This study aimed to evaluate the efficiency of the HIV surveillance system, which stands as a pivotal strategy in combating HIV/AIDS.

**Figure:**
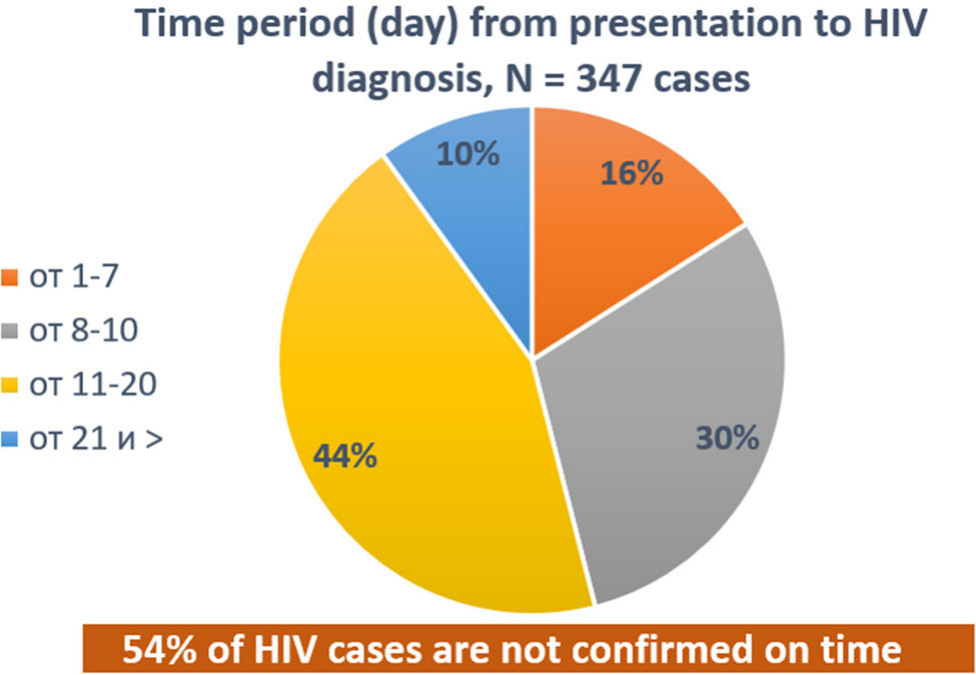

**Methods:**

Between February and March 2024, we conducted a descriptive evaluation study of the existing surveillance system using the CDC's methodology. The analysis covered data from the AIDS Centre and three health centers in Khujand, including a survey of 33 health workers to assess aspects of surveillance such as flexibility, timeliness, sensitivity, predictive positive value, cost, and overall effectiveness.

**Figure:**
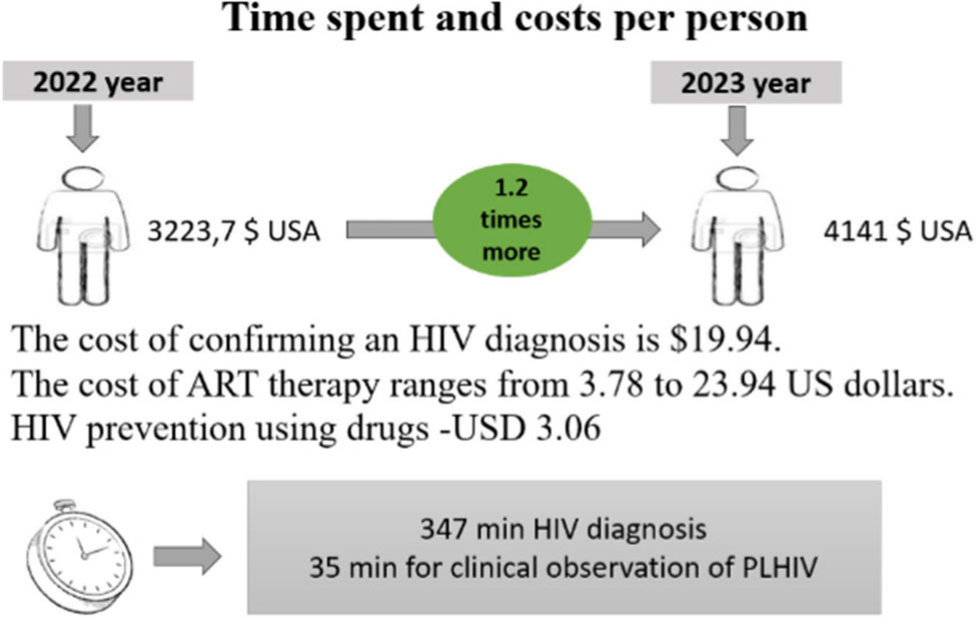

**Results:**

According to the decision of the Government of the Republic of Tajikistan, from 2012 to 2022, 12 AIDS centers were organized in the cities and districts of the region. However, to date, the infrastructure of these centres has not yet been fully developed. Also, 30% of commission blood samples are collected with a delay. The sensitivity of the system has reached 91%, which means that 91% of the estimated number of people living with HIV are detected by surveillance. The positive predictive value is 78%, indicating the probability that the reported case is indeed consistent with a real case of HIV. The cost of confirming an HIV diagnosis per person was $19.94. Also, the cost of antiretroviral therapy per month ranges from US$3.78 to US$23.94, which remains cost-effective, while HIV prevention costs US$3.06. In the region, HIV testing coverage increased by 1.2 times and reached 13.9% in 2023 compared to previous years, which led to a decrease in the mortality rate by 1% and indicates the effectiveness of the system.

**Conclusion:**

The system shows potential for further improvement and adaptation. Improved strategies need to be implemented to increase timeliness and flexibility in responses to epidemiological changes. Particular attention should be paid to optimizing logistics processes and expanding infrastructure to achieve higher standards in HIV diagnosis and treatment.

**Disclosures:**

**All Authors**: No reported disclosures

